# The Roles of Familiarity and Context in Processing Chinese *Xiehouyu*: An ERP Study

**DOI:** 10.1007/s10936-020-09753-0

**Published:** 2021-01-02

**Authors:** Xiaolu Wang, Yizhen Wang, Wanning Tian, Wei Zheng, Xiaoli Chen

**Affiliations:** 1 School of International Studies, Zhejiang University, No. 866, Yuhangtang Road, Hangzhou, 310058 People’s Republic of China; 2 School of International Studies, NingboTech University, Ningbo, People’s Republic of China; 3 School of Humanities and Communication Arts, Western Sydney University, Penrith, NSW Australia; 4 Willis Towers Watson Greater China, Shanghai, People’s Republic of China; 5 School of Foreign Languages, China Three Gorges University, Yichang, People’s Republic of China

**Keywords:** Chinese *xiehouyu*, Familiarity, Context, Event-related potentials (ERPs)

## Abstract

This study conducts an ERP experiment to explore the online processing mechanism of Chinese *xiehouyu,* a subcategory of Chinese idiomatic expressions with a metaphorical two-part allegorical saying, regarded as a non-literal language construct. Using a 2 × 2 design, (high familiarity (HF)/low familiarity (LF)) × (literally-biasing context (LC)/metaphorically-biasing context (MC)), the researchers have obtained the following findings: (1) familiarity plays an important role in Chinese *xiehouyu* processing, i.e. the metaphorical meaning of a HF Chinese *xiehouyu* can be directly activated while that of a LF one has to be derived from its literal meaning first; (2) contextual information also weighs in the process, i.e. the metaphorical meaning of a Chinese *xiehouyu* can be promoted in MC condition but suppressed in LC condition; (3) the interactive effect of familiarity and contextual information can be explained by the career of metaphor hypothesis; and (4) the Standard Pragmatic Model (SPM) of non-literal languages can explain the processing of LF *xiehouyu*, and the Direct Access Model (DAM) may to some extent account for the mechanism of HF one but fails to explain the case of LF one, while the Graded Salience Hypothesis (GSH) can provide an acceptable explanation for the processing mechanism of Chinese *xiehouyus* of varied familiarity.

**Supplementary information:**

The online version of this article (10.1007/s10936-020-09753-0) contains supplementary material, which is available to authorized users.

## Introduction

A Chinese *xiehouyu*, or a two-part allegorical saying, is a subcategory of Chinese idiomatic expressions. For instance, “粪坑里的石头——又臭又硬 (*Fèn kēng lǐ de shí tou* — *yòu chòu yòu yìng*)”, literally means “a stone in a manure pit—smelly and hard”, while carrying an implied metaphorical message of being “unpleasant and stubborn”. In this example, “a stone in a manure pit” is the antecedent, and “smelly and hard” is the consequent. The consequent literally portrays the features of the antecedent and can be mapped from “a stone” to human characteristics, figuratively describing the features of being “unpleasant and stubborn”. Through inference, the hearer may access the final interpretation that the person referred to has some negative characteristics of being “unpleasant and stubborn” (Shu 2015).

Just like this example, a Chinese *xiehouyu* is commonly made up of an antecedent and a consequent, most often linked by a dash or a comma in writing and separated by a pause in speaking. The antecedent is specific and vivid, usually portraying a realistic and/or imaginative object, situation or event, while the consequent is abstract and generalized, tending to identify certain attributes of the item or phenomenon in the first part. A mapping process is created from the antecedent to the consequent (e.g. Lai 2008; Ma and Zhang 2011; Zhang 2016). However, the mapping mechanisms are not the same for all Chinese *xiehouyus*, and *xiehouyus* can be further categorized as semantic *xiehouyus* and homophonic *xiehouyus* according to how their non-literal meanings are constructed through varied ways of mapping (Zhang et al. 2013, 2018; Ma et al. 2019): The figurative meaning of the former is activated by metaphorical mapping, while that of the latter is achieved by phonological mapping.

The example mentioned above “粪坑里的石头——又臭又硬 (A stone in a manure pit—smelly and hard)” is a semantic *xiehouyu*, in which the two parts have a strong semantic association. The antecedent and the consequent are related literally, and when they co-occur, they together convey a non-literal meaning metaphorically. The features of the antecedent being “smelly and hard” is mapped to the target domain—“human characteristics” which shares with the source domain the features of being “unpleasant and stubborn”. As for homophonic *xiehouyus*, the semantic connection between the antecedent and the consequent is not obvious, while the meaning of the *xiehouyus* is accessed through homophonic puns in the consequent.

For example, “外甥打灯笼——照舅/照旧 (*wài sheng dǎ dēng long*——*zhào jiù/zhào jiù*)” means “a nephew is holding a lighted lantern——illuminating his uncle/as before”. The reason we can activate the figurative meaning “as before” is that “舅 (uncle)” and “旧 (old)” share the same pronunciation “*jiù*”, and when “照” and “旧” combine as the word “照旧”, it means “as before”. Therefore, homophones act as a medium to activate the non-literal meaning through sound association. Between these two types, semantic *xiehouyus* account for a significantly larger proportion (almost 90%), and more high familiarity items can be found in this category than in the homophonic category (Zhang 2016: 185), thus the researchers can pick more suitable experimental materials from semantic *xiehouyus*. In this research, we focused only on the processing mechanism of semantic *xiehouyus*.

Unlike other types of non-literal languages (e.g. metaphors, humors, puns, idioms, ironies) which have only one part, a Chinese *xiehouyu* has a unique two-part structure, making them worthy of study. As for those one-part, non-literal constructs, their figurative meaning is implied in the literal body, while a *xiehouyu* visually shows both an explicit literal meaning (the antecedent) and an extended non-literal meaning (the precedent). But a *xiehouyu* has not been studied as much as other figurative language forms. Investigating into *xiehouyus* can not only help people know more about this special non-literal language construct, but also contribute to the overall study of non-literal language processing mechanisms and the development of related theories.

For the processing of figurative languages, researchers tend to consider whether their literal meaning should be activated first, or whether the figurative meaning can also be processed directly. Among the related theories, the **Standard Pragmatic Model (SPM)**, the **Direct Access Model (DAM)** and the **Graded Salience Hypothesis (GSH)** seem to be the prevailing theories.

The SPM of non-literal language comprehension is derived from the pragmatic theories in the 1970s to 1980s (Grice 1975; Searle 1979), suggesting that the literal meaning of expressions would always get accessed prior to the non-literal meaning, and the latter is to take place only when the incongruity between the literal meaning and contextual information is noticed (Giora 1999). Contrarily, the DAM claims a direct access to non-literal interpretation which may be revealed by no significant time delay compared to literal processing (Gibbs 1984, 2002), and it is the contextual information that plays the decisive part. However, scholars often find fault with the DAM given either differences in processing familiar and unfamiliar non-literal languages or longer reading times for non-literal expressions than for equivalent literal ones (Giora 1997).

In the light of the roles played by encapsulated linguistic information as well as contextual information in non-literal language comprehension proposed by the SPM and the DAM, the GSH breaks the dichotomy of literal and non-literal meaning, and puts forward an ordered lexical access mechanism, i.e. more salient meanings occur to people first due to conventionality, frequency, familiarity or prototypicality, being accessed and activated prior to less salient ones, regardless of contextual information or authorial intent (Giora 2003). According to the GSH, contextual information only influences the final product of language processing, while salience is the decisive factor to activate the real meaning of the expression. In this study, we try to find out which of the three theories is more suitable for explaining the processing of *xiehouyus*, and how cognitive processing of *xiehouyus* can provide more insights for these theories.

Many factors influence the processing of figurative languages: internal factors such as familiarity and concreteness of the items, and external factors like linguistic or non-linguistic contexts. Among them, the effects of familiarity and contextual information have attracted the most attention from scholars. Familiarity is a subjective factor influencing cognitive processing (Wang and Xu 2008). Different from frequency, familiarity is based on personal language experience rather than merely on the frequency of appearance in public language use. Giora (1997) proposed that familiarity is one of the major factors determining the saliency of meaning, that the non-literal meaning of highly familiar non-literal languages is more salient than or equally salient to their literal meaning, and that the salient meaning of the less familiar ones should be their literal meaning.

The important role played by familiarity has been observed in a series of empirical studies on non-literal language processing, and most of them found that familiar expressions require less efforts to process than unfamiliar ones (e.g. Schweigert and Danny 1988; Titone and Connine 1999; Lim et al. 2009; Titone and Libben 2014). Particularly, the effect of familiarity has also been reported by several research studies on Chinese *xiehouyus*. Qu (2010) discovered that reading highly familiar Chinese *xiehouyus* in metaphorically-biasing contexts tends to be significantly faster than reading unfamiliar ones. Ma and Zhang (2011) claimed that familiarity has positive correlations with predictability, comprehensibility, concreteness and coherence of Chinese *xiehouyus*, and that the consequent of a highly familiar Chinese *xiehouyu* tends to be easier to predict from its antecedent. In other words, familiarity is the predominant factor to determine if a Chinese *xiehouyu* can “*xiehou*” i.e. drop the consequent when it is in use, because only those consequents of highly familiar Chinese *xiehouyus* could be omitted (Zhang et al. 2018). Generally, highly familiar expressions can be processed much faster with less cognitive effort.

As for the effect of context, it can be described in two aspects: (1) the time when contextual information comes into play; (2) how different kinds of contextual information function. Firstly, the three processing models of non-literal languages (the SPM, DAM, and GSH) mentioned in the second paragraph have been developed with various opinions on the temporal stage when contextual information takes effect. Both the SPM and the GSH believe that context does not work in the early stage of processing, and literal meaning is always activated and processed first, while the DAM argues that context plays a role in the beginning of lexical activation, and the meaning consistent with context is directly extracted.

Secondly, studies on non-literal languages claim that metaphorical context primes the expression’s metaphorical meaning, and literal context promotes the item’s literal meaning (e.g. Katz and Ferretti 2001; Wang et al. 2018). But the GSH argues that if a meaning is salient enough, its activation is hardly influenced by the context. Until now we have not found a published paper that provides empirical evidence for the contextual effect on *xiehouyu* processing. Therefore, although studies are providing accumulating evidence supporting the essential role of familiarity on *xiehouyus*, or non-literal language processing, the results on the contextual effect are still in dispute, and the interaction between familiarity and context is not yet clear.

Not many studies on Chinese *xiehouyus* have been done with empirical approaches so far. We have only found that several scholars used a questionnaire survey (Qu 2010), corpus (Ma and Zhang 2011), eye tracking (Ma et al. 2019) and/or neuropsychological experiment (Zhang et al. 2013) to explore the dynamic comprehension mechanism of Chinese *xiehouyus* as well as to examine the conclusions drawn from theoretical analyses. Among them, Zhang et al.’s study (2013) is the first and only one published using event-related potential (ERP) technique to study Chinese *xiehouyus*, and their results show that highly familiar Chinese *xiehouyus* can experience a direct access to metaphorical meaning, while the metaphorical meaning of unfamiliar ones can only be comprehended after the literal meaning is understood by the receiver. However, Zhang’s study simply took the antecedents as literally-biasing contexts, but did not pay attention to the effect of external contexts. As Chinese *xiehouyus* are seldom used independently without linguistic contexts, it is important to explore how this kind of language form is comprehended when certain contextual information is provided.

To explore the roles of familiarity and context on the online processing mechanism of *xiehouyus*, we used the ERP technique, which can facilitate further observations on the brain activities during language comprehension. The ERP technique is derived from electroencephalography (EEG), a widely used brain imaging technique measuring the postsynaptic neural activities of large neural populations in the millisecond range. ERPs are time-locked neural responses associated with specific sensory, cognitive, and motor events, extracted from the continuous EEG by means of averaging (Luck 2005: 4). With very high temporal resolution, ERP enables us to trace the very rapid processes involved in language comprehension in our brain, and it allows random representation of stimuli across conditions, facilitating the comparative studies between conditions.

N400 is a well-known ERP component, a centroparietal negativity peaking at around 400 ms after the onset of the critical stimuli. It was first discovered as an indicator of semantic violations or anomaly (Kutas and Hillyard 1980), but later many studies argue that N400 reflects more about predictability than semantic incongruity (e.g. Kutas and Federmeier 2011; Lau et al. 2016). In all cases, the related items which were more predictable elicited reduced N400 amplitudes compared to unrelated ones across different tasks. The amplitude of N400 is therefore focused in this study to reveal the contextual predictability between the priming context and Chinese *xiehouyus* with varied degrees of familiarity, as well as the amount of cognitive effort required in the process.

From above, we know that there exist research gaps in this topic. First and foremost, until now we have only found one study concerning the neuro-cognitive mechanism of Chinese *xiehouyus* using ERP technique. Therefore, this study can provide more insight into Chinese *xiehouyu* processing. Moreover, as introduced before, the roles of familiarity and contextual information on non-literal language processing have often been investigated separately but actually they often play their roles interactively. This paper moves forward to their potential interactions, trying to explore the discrepancies between the processing mechanisms in semantically-biasing contexts of Chinese *xiehouyus* varied in different degrees of familiarity. In addition, the research also attempts to compare the explanatory power of the prevailing processing models of non-literal languages. In a nutshell, we plan to answer the following questions:

1. What roles do familiarity and linguistic context play respectively and interactively in the processing of Chinese *xiehouyus*?
2. Which of the prevailing models for non-literal language processing, namely the SPM, DAM and GSH, is able to give a more satisfactory account for the processing mechanism of Chinese *xiehouyus*?
3. Whether the antecedent of a Chinese *xiehouyu* can be used independently, or if it has to be used with the consequent?

## Methods

### Experiment Design

This study is basically an extension of Zhang et al. (2013)’s, the first attempt to observe the online processing mechanism of highly familiar and unfamiliar semantic Chinese *xiehouyus* with ERP technique in a riddle-guessing paradigm. Our study, however, takes one step further to observe the online processing of semantic Chinese *xiehouyus* not only from the role played by familiarity, but also from the role of context, based on the time course in an ERP experiment. A Prime-Target-Probe paradigm was adopted with two-character words biasing either the literal or metaphorical interpretation of Chinese *xiehouyu*s serving as the priming context, and with the antecedents as Targets and the consequents as Probes.

By analyzing the ERP results on the Targets, we detected how the meaning of antecedents can be accessed from the preceding **literally-biasing context (LC)** or the **metaphorically-biasing context (MC)**. The models of figurative language processing mechanism of SPM, DAM and GSH will be examined against their different assumptions for the results. Generally speaking, both for Chinese *xiehouyus* with high or low familiarity, the SPM predicts a smaller N400 amplitude and a better behavioral performance in the processing primed by LC condition than MC condition. As the SPM model suggests that the access of literal meaning is mandatory, that the activation of metaphorical meaning can take place only after the denial of literal meaning and that the access of metaphorical meaning always requires more cognitive efforts, we can infer that it is more difficult and time-consuming to activate the metaphorical meaning regardless of familiarity of a Chinese *xiehouyu* and the context in which it lies.

On the other hand, the DAM suggests that either the literal or non-literal meaning supported by the corresponding context will be primed, which might predict no clear significance between MC and LC condition in terms of ERP and behavioral results. The third model, the GSH, however, claims a parallel mechanism for different meanings in language processing, and predicts a faster and easier access to the salient metaphorical meaning of HF ones, as well as a successful access to the less salient literal meaning in LC condition. The ERP results on the Probes and behavioral results will mainly inform us about the difficulty in semantic association between the two parts of a Chinese *xiehouyu*, on which the effect of familiarity may be prominent, but we are not sure whether the continuous effect of context can be observed.

### Participants

Thirty undergraduate and postgraduate students (25 females, 5 males; mean age = 19.5 ± 1.6) participated in the experiment with monetary compensation, all of whom were right-handed native Chinese speakers, with normal or correct-to-normal vision. None of them reported any history of neurological or psychiatric disorders. ERP data from five participants were discarded due to excessive artifacts in EEG recordings, and behavioral data from another two participants were removed due to bad results (behavioral accuracy < 75%). Informed consent was obtained from all individual participants included in the study. All procedures performed in studies involving human participants were in accordance with the ethical standards of the Research Ethics Committee and with the 1964 Helsinki declaration and its later amendments or comparable ethical standards and complied with the specific requirements of the country.

### Stimuli

At first, 106 semantic Chinese *xiehouyus* were selected from *Dictionary of Chinese Xiehouyus* (Wen 2011) as the materials for the pretest. They are all made up of an antecedent with the length of 4–5 Chinese characters and a consequent with 2–4 Chinese characters. A group of 40 undergraduates and postgraduates who did not participate in the ERP experiment was given a five-point Likert scale (5 for highly familiar and 1 for highly unfamiliar) to test the familiarity of the 106 Chinese *xiehouyus*. As a result, we obtained 80 out of 106 semantic Chinese *xiehouyus* for the formal testing, 40 of which were with **high familiarity (HF)**, rated ≥ 4 by 80% participants (*MD *= 3.89 ± .46), and the other 40 were with **low familiarity (LF)**, rated ≤ 2 by over 80% participants (*MD *= 1.27 ± .29, *t *= 16.586, *p *< .001). A **literally-biasing two-character word context (LC)** as well as a **metaphorically-biasing one (MC)** were then designed referring to the literal and the metaphorical interpretation of the 80 Chinese *xiehouyus*, getting 160 paired “Prime (two-character word context)—Target (antecedent)—Probe (consequent)” materials in total. These contexts were supposed to serve as the minimum priming contexts in the ERP experiment. For example, a literally-biasing context “手感 (hand feel)” and a metaphorically-biasing context “为人 (behavior style)” were designed for the Chinese *xiehouyu* “皮球抹油——又圆又滑”, of which the antecedent means “a greased ball” and the consequent after the dash means literally “being round and slippery”, but metaphorically “slick and sly”. To test the effectiveness of contexts, another 40 participants from the same subject population but not participating in the ERP experiment were recruited and evenly divided into two groups. They were asked to rate either the relatedness between the literal meaning of Chinese *xiehouyus* and their LC, or the relatedness between the metaphorical meaning of Chinese *xiehouyus* and their MC on a five-point Likert scale (5 = highly associated, 1 = unassociated). Based on the results, the researchers made minor revisions of the context. The mean scores of the relatedness between the LC and the literal meaning of Chinese *xiehouyus* was 4.19 ± .48, and 4.16 ± .43 between the MC and the metaphorical meaning of the *xiehouyus* (*t* test, *p *> .1).

The 160 selected Prime-Target-Probe materials then went into two lists with 80 trials for each. Latin Square design was adopted to avoid repetition effects, that is, each Target-Probe pair occurred only once in one list but was paired with a different prime in another. In this way, each list contained four types of trials standing for the four conditions in this experiment, namely sentences of 20 **Type One (T1) **= high familiarity with a literally-biasing context **(HFLC)**, 20 **Type Two (T2)** = low familiarity with a literally-biasing context **(LFLC)**, 20 **Type Three (T3)** = high familiarity with a metaphorically-biasing context **(HFMC)**, and 20 **Type Four (T4)** = low familiarity with a metaphorically-biasing context **(LFMC)** as the stimuli (see Table 1).

**Table 1:** Sample stimulus trials used in the ERP experiment

Condition	Stimulus trials		
	Prime	Target	Probe
HFLC	庄稼 (crop)	芝麻开花 (sesame blooming)	节节高 (to grow higher and higher)
LFLC	饭菜 (dish)	白水煮豆腐 (boiling tofu in plain water)	淡而无味 (tasteless)
HFMC	事业 (career)	芝麻开花 (sesame blooming)	节节高 (to promote to a higher and higher position)
LFMC	文章 (article)	白水煮豆腐 (boiling tofu in plain water)	淡而无味 (boring)
Filler	犯罪 (crime)	老鼠拉王八 (a mouse pulling a tortoise)	难回家 (hard to go home)

Due to the restrictions of the fact that Chinese *xiehouyus* applicable for this experiment were uneven in length and limited in number, it was impossible to uniform the lengths of all the Target and Probe items. However, to minimize the potential interference produced by character length, the lengths of Targets and Probes were controlled among the conditions. In both lists, no significant difference (one-way ANOVA, *p *> .1) of the average length of Targets and Probes separately existed among the four types of trials. **80 filler trials** resembling the critical trials in form were also included in each list. The pairs of Target-Probe fillers were made up of pseudo-*xiehouyus* in which a paired antecedent part and the consequent part were not semantically related to each other, and the two-word context and the paired Chinese *xiehouyu* had no semantic associations, either. Both of the testing lists used the same filler trials. For each participant, the 80 trials in one testing list were pseudo-randomly intermixed with the 80 filler trials.

### Procedure

**Fig. 1:**
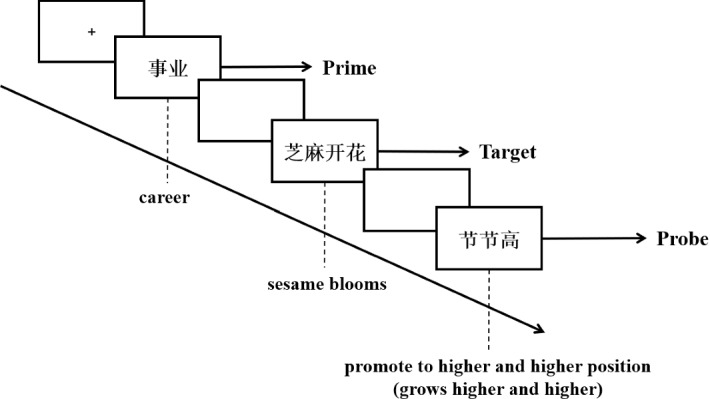
Flowchart of a trial

Each participant was required to sit in a dimly-lit and sound-attenuated room facing a computer screen about 1.2 m away, resting his/her index fingers on the F and J keys of a keyboard. Before the experiment started, each participant was informed to read carefully the instructions highlighting all the dos and don’ts (e.g. fixate on the central cross every time it appears, and refrain from eye and body movement, etc.) word by word and communicate with the tester if he/she had any questions. All stimuli were presented one by one on the computer screen with the background color being black and the foreground being white, and the presentation was controlled by E-Prime 2.0. The participants were only told that they would read some trials made up of Chinese phrases, and need to decide whether or not the Probe was semantically related to the Target by pressing the “F” key or the “J” key on the keyboard. Key-hand mapping was counterbalanced across the participants. The participants were also given a brief practice session to get familiar with the task and procedure. In the practice and experiment sessions, each trial started with a white fixation cross at the center of screen. The Prime was then presented for 800 ms, followed by a blank screen lasting for 500 ms. After that, the Target was presented for 1300 ms, and again followed by a 500 ms blank screen. The Probe then appeared and stayed on the screen until the participant made judgment on whether the Target and Probe were semantically related through the key response (see Fig. 1). The testing session consisted of three blocks and lasted around 20 min in total. Before the formal experiment, six practice trials were provided for the participants to have a better grasp of the procedures.

### EEG Recording

EEGs were continuously recorded with an elastic 32-channel cap from Brain Products Inc (BP). The electrode sites were following the extended 10–20 convention. The vertical Electroencephalograph (VEOG) was recorded with the electrode placed below the right eye. The horizontal Electroencephalograph (HEOG) was recorded with the electrode placed near the outer corner of the right eye. Electrode impedances were kept below 5 kΩ, and the amplified analog voltages were sampled at 1000 Hz with .05–100 Hz bandpass. The EEGs were analyzed with BP Analyzer. The signals were filtered at the level of low pass 30 Hz, zero phase shift and high pass .1 Hz, zero phase shift. After that, ocular and movement artifacts were removed in a semi-automatic manner. For ocular artifacts, the parameters were set at 20 for a minimum number of sweeps, 400 for blink duration, and 10% for the threshold. Average ERP results free of ocular and movement artifacts from − 200 to 1000 ms after the onset of the Target and the Probe were computed with the first 200 ms as the baseline and then computed by subjects. Based on this, the grand average ERPs of each condition (HFLC, LFLC, HFMC and LFMC) were obtained. As mentioned in the Participants Section, data from 2 participants were excluded from behavioral and ERP result analysis due to bad response accuracy. Additionally, another 5 participants’ ERP data were not involved in the ERP grand total due to excessive artifacts.

## Results

### Behavioral Results

We reported the accuracy (Acc.) and the response time (RT) of different conditions in this part (see Table 2). In terms of response accuracy, a main effect of familiarity (*F* (1, 27) = 15.183, *p *< .001, *η*_*p*_^*2*^ = .360) was able to be detected. To be more specific, the rate of response accuracy of HF items was significantly higher than that of the LF ones (98.3% vs. 95.5%). We found no significant main effect of context type (*F* (1, 27) = 2.065, *p *> .1), but the interaction between context type and familiarity was significant (*F* (1, 27) = 6.242, *p *= .019, *η*_*p*_^*2*^ = .188). Planned comparisons showed that in the MC condition, participants made significantly more correct judgments on HF *xiehouyus* than LF *xiehouyus* (98.5% vs. 94.1%, *t *= 4.459, *p *< .001, two-tailed), but not in the LC condition (98.0% vs. 96.8%, *t *= 1.216, *p *> .1, two-tailed). Meanwhile, a significant effect of context was revealed from LF *xiehouyus*: response accuracy of LF *xiehouyus* preceded by LC condition was significantly higher than that of those preceded by MC condition (96.8% vs. 94.1%, *t *= 2.526, *p *= .031, two-tailed), but no context effect existed in HF ones (98.0% vs. 98.5%, *t *= − .757, *p *> .1, two-tailed).

**Table 2:** Behavioral results

Condition	Acc. (%)	RT (ms)
HFLC	98.0 ± .285	725.471 ± 171.330
LFLC	96.8 ± .291	1185.404 ± 368.630
HFMC	98.5 ± .344	727.336 ± 187.355
LFMC	94.1 ± .584	1138.025 ± 329.787
Filler	84.8 ± .371	1402.341 ± 320.145

RTs to the Probes of 28 participants in the correct trials were firstly trimmed: those below 200 ms (.06%) or deviated 2.5 interquartile range (.88%) were removed. Then statistical analyses carried out on RTs revealed a main effect of familiarity (*F* (1, 27) = 59.714, *p *< .001, *η*_*p*_^*2*^ = .713), so that the participants were responding to LF *xiehouyus* (1161.715 ms) more slowly than they were responding to HF *xiehouyus* (726.404 ms). However, there was neither main effect of context (*F* (1, 27) = .021, *p *> .1,) nor interaction between the two factors (*F* (1, 27) = 2.258, *p *> .1).

Besides, we compared the HF condition and the LF condition with the filler condition in terms of familiarity, since the filler condition can serve as a good baseline for the LF condition. The accuracy of the filler condition was significantly lower than that of the LF condition (84.8% vs. 95.5%, *t *= − 7.167, *p *< .001, two-tailed) and the HF condition (84.8% vs. 98.3%, *t *= − 9.043, *p *< .001, two-tailed). The RTs of the fillers were also significantly longer than that of the LF condition (1402.341 ms vs. 1161.715 ms, *t *= 6.334, *p *< .001, two-tailed) and the HF condition (1402.341 ms vs. 726.404 ms, *t *= 17.790, *p *< .001, two-tailed).

### ERP Results

**Fig. 2:**
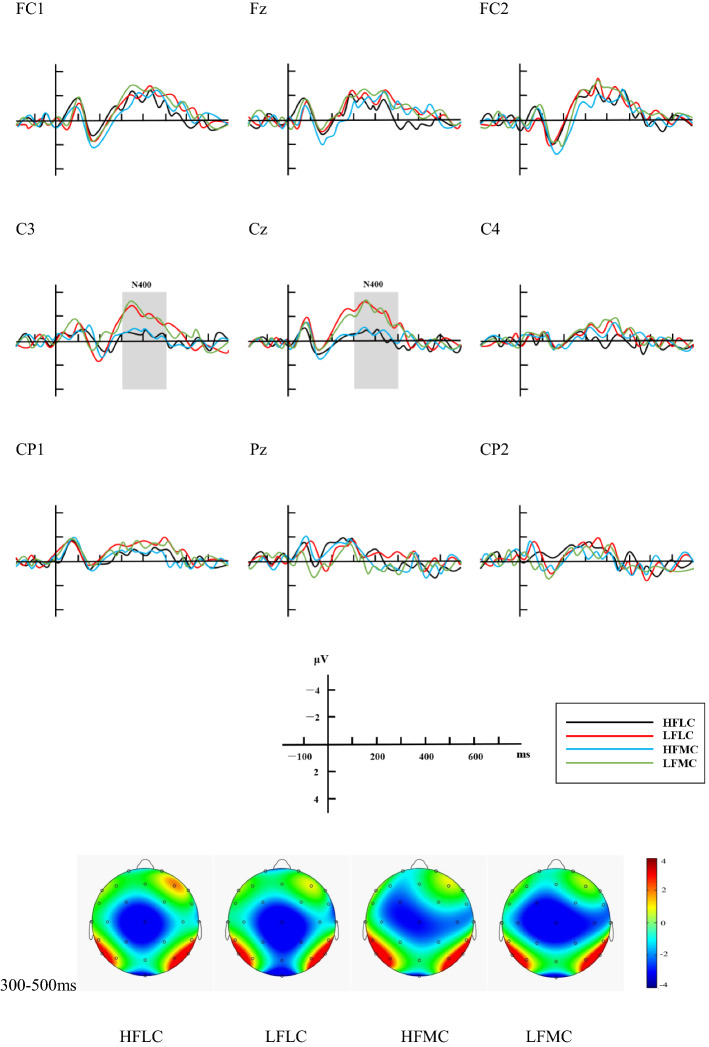
Grand average ERP response to the targets for four conditions

**Fig. 3:**
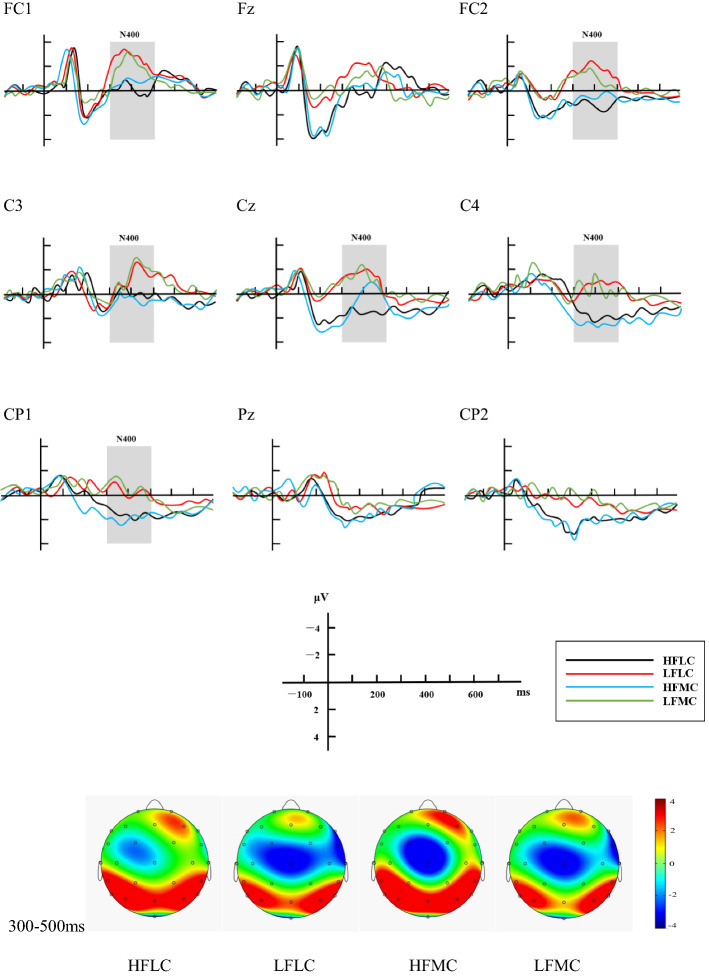
Grand average ERP response to the probes for four conditions

As for the ERP responses to the Targets and Probes, statistical analyses were performed on the time windows of 300–500 ms after the stimuli (both the Targets and the Probes) appeared, to capture the N400 effects (FC1, C3, CP1, Fz, Cz, Pz, FC2, C4 and CP2 as nine representative electrodes, see Figs. 2, 3). The amplitude of the N400 component from the electrodes mentioned above were then analyzed through a three-way repeated-measure ANOVA, including 2 familiarity levels (HF, LF) × 2 context types (LC, MC) × 3 conditions of laterality (left (L): FC1, C3, CP1; middle (M): Fz, Cz, Pz; and right (R): FC2, C4, CP2).

In terms of the ERP results regarding the Targets, we found a main effect of familiarity (*F* (1, 68) = 8.055, *p* = .006, *η*_*p*_^*2*^ = .106): the mean amplitude of N400 elicited by HF *xiehouyus* was significantly less negative than that elicited by LF ones (− .568 µV vs. − .804 µV). But no main effects of context type (*F* (1, 68) = 2.339, *p* > .1) and laterality (*F* (2, 136) = 1.897, *p* > .1) were revealed. There was an interaction between familiarity and context type (*F* (1, 68) = 15.806, *p* < .001, *η*_*p*_^*2*^ = .189). Planned comparisons demonstrated that the mean amplitude in the HFMC condition was significantly less negative than that in the LFMC condition (− .326 µV vs. − .886 µV, *t *= 4.118, *p *< .001, two-tailed) and in the HFLC condition (− .326 µV vs. .809 µV, *t *= 3.554, *p *< .001, two-tailed). No interactions were revealed between familiarity and laterality (*F* (2, 136) = 1.136, *p* > .1), between context type and laterality (*F* (2, 136) = 1.068, *p* > .1), or among the three factors (*F* (2, 136) = 0.187, *p* > .1).

In addition, we also compared the HF condition and the LF condition with the filler condition to explore more about the familiarity, as we explained in the section of behavioral results. The ERP results presented a stronger N400 effect after the onset of Targets in the filler condition than in the LF condition (− 1.223 µV vs. − .804 µV, *t *= − 3.082, *p *< .001, two-tailed) and the HF condition (− 1.223 µV vs. − .568 µV, *t *= − 4.820, *p *< .001, two-tailed).

For the ERP results of the Probes, ANOVA revealed a significant main effect for familiarity (*F* (1, 68) = 17.330, *p *< .001, *η*_*p*_^*2*^= .203): the mean N400 amplitude elicited by HF consequents was significantly less negative than that of LF ones (.703 µV vs. .171 µV). There were no main effects of context type (*F* (1, 68) = .46, *p *> .1) and laterality (*F* (2, 136) = .136, *p *> .1). Additionally no interactions were revealed between familiarity and context (*F* (1, 68) = .088, *p* > .1), between familiarity and laterality (*F* (2, 136) = .164, *p* > .1), between context and laterality (*F* (2, 136) = .198, *p* > .1), or among the three factors (*F* (2, 136) = 1.158, *p* > .1).

The N400 effect elicited in filler condition was also compared with that in the HF condition and the LF condition after the onset of Probes. The N400 amplitude in the former was significantly larger than that in the LF condition (− .283 µV vs. .171 µV, *t *= − 3.340, *p *< .001, two-tailed) and the HF condition (− .283 µV vs. .703 µV, *t *= − 7.253, *p *< .001, two-tailed).

## Discussion

Based on the data collected from the ERP experiment above, in this section we try to answer “What roles do familiarity and linguistic context play respectively and interactively in Chinese *xiehouyu* processing?” in the first part, “Which of the prevailing models of non-literal language processing, namely the SPM, DAM and GSH, is able to give satisfactory accounts for the processing mechanism of Chinese *xiehouyus*?” in the second part, and “Whether the antecedent of a Chinese *xiehouyu* can be used independently or if it has to be used with the consequent?” in the last part.

Before starting the discussion, it is necessary to explain what can be learned from the ERP results and the behavioral results. The ERP results obtained for the Targets (antecedents) demonstrate the meaning activation process as well as the difficulty of associating with the preceding contexts. The results obtained for the Probes show the difficulty of semantic associations between the antecedents and consequents. Moreover, it is necessary to point out that behavioral data display the participants’ performance in the semantic judgment tasks (i.e. to identify whether the Probes are semantically related to the Targets). They can to some extent indicate the ultimate results of the comprehension process, but are not sensitive enough to the real-time status in processing. In addition, the ERP and behavioral results of the comparisons between the filler condition, LF condition and HF condition also help explain the role of familiarity on comprehending Chinese *xiehouyus*, because the filler condition can be regarded as a good baseline for the LF condition.

## The Respective and Interactive Effects of Familiarity and Context

### The Respective Effect of Familiarity and Context

1. The Effect of Familiarity

The effect of familiarity can be detected from the ERP results of both the Targets and the Probes, as well as the behavioral results. First, looking at the ERP results obtained for the Targets (the antecedents), the HF antecedents had significant processing advantages over the LF antecedents (− .568 µV vs. − .804 µV, *p* = .006). This pattern has been reported in a series of previous studies on non-literal language processing (e.g. Giora and Fein 1999; Katz and Ferretti 2001; Qu 2010; Zhang et al. 2013), indicating that familiarity is a crucial factor here. As for HF antecedents, participants accessed their meaning more quickly, and associated them with the preceding contexts more easily. Thus, the N400 effect was weaker than that induced by LF antecedents. Furthermore, though LF antecedents were not that familiar to the participants, the phrases could still be predicted from the priming contexts, unlike the fillers with no sense, which can be observed from the ERP responses to the Targets that the filler condition elicited significantly stronger N400 effects than the LF ones (− 1.223 µV vs. − .804, *p *< .001). The reason is that the two-character words of context in fillers had nothing to do with the pseudo-antecedents, but LF antecedents were semantically related to the MC or LC, despite the fact that the link was harder and slower to activate than that of HF ones.

For the Probes, HF consequents only elicited very small and localized N400 effects (see Fig. 3), indicating a fairly easy cognitive process. It can be inferred from such results that HF Chinese *xiehouyus*, just like other HF formulaic sequences, have been previously stored as chunks in long-term memory, so that when the antecedents were presented to the participants, they had strong expectations for the consequents on the basis of former language experience, and even activated the lexical structure of the consequents earlier than their appearance. As a result, the participants only needed to check whether the presented HF consequents were in accord with what they had retrieved from long-term memory, but further reasoning processes seemed less necessary. In the case of LF Chinese *xiehouyus* after the onset of Probes, however, the robust and widespread N400 waveform (see Fig. 3) might indicate that the semantic relatedness between the two parts had not been previously established in mental lexicon, and the participants failed to make correct predictions on the consequences from the priming contexts and the antecedents (Zhang 2016: 198). As a result, cognitive efforts including logical deduction and creative thinking processes might be involved to shift from the literal meaning of LF antecedents to the metaphorical meaning of the consequents via metaphorical thinking (Lai 2008). However, though it is hard to establish the semantic relations between the two parts in LF Chinese *xiehouyus*, the participants could still perceive such an association, which is proved by the weaker N400 effects elicited by LF conditions than filler conditions after the onset of Probes (.171 µV vs. − .283 µV, *p *< .001). When the participants saw the unrelated two parts of the fillers, they would feel more surprised than when they saw that of the LF ones. This phenomenon shows that LF Chinese *xiehouyus* can be understood like other kinds of non-literal languages, but need longer processing time compared to the HF ones.

The effect of familiarity was also clearly revealed by the accuracy and RT results, as it took significantly longer time for the participants to comprehend LF Chinese *xiehouyus* than HF ones (1161.715 ms vs. 726.404 ms, *p *< .001), and the participants made more wrong judgements on LF ones than HF ones (95.5% vs. 98.3%, *p *< .001), replicating the RT results in past experiments investigating the familiarity effect on Chinese *xiehouyus* as well as sentential idiomatic expressions (Katz and Ferretti 2001; Zhang et al. 2013). Though the subjects behaved worse in LF *xiehouyus* than in HF items, they could still comprehend the LF items, since the behavioral data of the LF ones were much better than that of fillers with non-semantic relatedness (*p *< .001 in terms of accuracy and RT).

To sum up, the effect of familiarity can be detected at different stages in the processing of Chinese *xiehouyus*. Familiarity is a significant factor for meaning activation, associating with preceding contexts and dominating the predictability of consequents. The processing can be completed for both HF and LF Chinese *xiehouyus*, though it is harder to reach the non-literal meaning of LF ones.

(2) The Effect of Context

There were no main effects of context in ERP results for the Targets and Probes, nor in behavioral data (all *p* > .1), so the next part “The Interactive Effect of Familiarity and Context” further analyzes the effect of context based on the interaction between familiarity and context shown in the data of Target and accuracy.

But in this part, we can describe the effect of context briefly. If we see from a general point, context actually plays a part in Chinese *xiehouyus* of both low and high familiarity. Based on ERP data on the Targets, we can find the influence of context in HF Chinese *xiehouyus*: the metaphorical meaning of the antecedents was quickly activated, promoted by the preceding MC condition but suppressed by LC condition (.809 µV vs. − .326 µV, *p* < .001). And from the behavioral results of accuracy, we can get a clue that context also works in LF Chinese *xiehouyus*. Because participants were not familiar with those LF *xiehouyus*, the antecedents could lead to literally-biasing interpretation rather than activate the metaphorical meaning directly. Then, compared to the LC condition, the MC condition might conflict with the literal meaning, and to some extent restrict the processing and cause mistakes (96.8% vs. 94.1%, *t *= 2.526, *p *= .031). More explanations of context are recorded in the next section.

### The Interactive Effect of Familiarity and Context

The interactive effect of familiarity and context can be explored from ERP data on Targets and Probes, and from behavioral results as well. Based on that, we find that it is suitable to explain the effect by the career of metaphor hypothesis (Bowdle and Gentner 2005). The career of metaphor hypothesis offers a unified theoretical framework that can resolve the debate between comparison and categorization models of metaphor. This account further suggests that whether metaphors are processed directly or indirectly depend on their degree of conventionality.

The antecedents of semantic Chinese *xiehouyus* can be regarded as the vehicle in a metaphorical structure (Lai 2008; Shu 2015). The LF antecedents are actually vehicles of new (novel) metaphors, which, according to the career of metaphor hypothesis, is at the first stage of the metaphor career. Their metaphorical meaning must be acquired through an ad hoc process of conceptual mapping from the vehicle to the topic because only their literal meanings are familiar to people. In this experiment, the accuracy rate of processing LF Chinese *xiehouyus* in MC conditions was significantly lower than in LC conditions (94.1% vs. 96.8%, *p *= .031). The reason for this might be that the meaning of LF *xiehouyus* was more literal than metaphorical for most participants. If primed by a MC condition, the contextual information remaining in working memory might conflict with the prevailing literal meaning of LF antecedents, thus leading to poor comprehension results.

If frequently used, novel metaphors can move on to the second phase in the metaphor career and become conventional. During such a period, the HF antecedents serve as metaphor vehicles with dual reference: both literal and metaphorical (Gong and Zhou 2009). Moreover, Bowdle and Gentner (2005) claimed that metaphors may develop from being “conventional” to “dead” at the end of the career. At that time the literal meaning of the metaphor vehicles dies out, leaving only the abstract metaphorical meaning. In this sense, the reason why the HF antecedents have more processing advantage in MC conditions than LC conditions (.809 µV vs. − .326 µV, *p *< .001) can be interpreted as actual vehicles at a transition state between being “conventional” and “dead”, when the metaphorical meaning prevails over the evanescent literal meaning. The metaphorical meaning of HF antecedents prevails over the literal meaning and can be quickly activated, and this metaphorical meaning can be promoted by the preceding MC condition. Thus, the participants felt the HF antecedents more metaphorical than literal. This finding is also consistent with the results reported in Katz and Ferretti (2001) that HF proverbs were read faster in MC conditions than in LC conditions. In other words, the metaphorical meaning of HF antecedents might be directly activated through a lexical process rather than derived from the correlating literal meaning. Therefore, processing HF antecedents in LC conditions are more difficult than in MC conditions, for literal contextual information suppresses the prevailing but incongruent metaphorical meaning, while promoting the processing of the congruent but shaded literal meaning. There is also additional evidence from the ERP results on Targets that HF antecedents were processed more easily than the LF ones in MC conditions (− .326 µV vs. − .886 µV, *p *< .001), implying the quick access to the metaphorical meaning of the HF stimuli (e.g. Giora and Fein 1999; Qu 2010).

Generally, the interactive roles of familiarity and context can be explained by the career of metaphor hypothesis. HF *xiehouyus* are vehicles of conventional metaphors that their metaphorical meaning prevails over literal meaning in processing and can be better accessed from metaphorical context. Besides, LF *xiehouyus* can be regarded as the vehicles of novel metaphors, whose metaphorical meaning has to be inferred from the literal meaning rather than be activated directly and can be predicted more quickly from literal context.

### The Non-literal Language Processing Models and Chinese *Xiehouyu* Processing

This study may also shed some light on the long-lasting debate on the temporal stage when the figurative meaning of non-literal language is activated, according to the processing of *xiehouyus*. In this section, the results obtained in this research are reexamined from the points of view of SPM, DAM, and GSH.

### The Standard Pragmatic Model (SPM)

As introduced by Grice (1975) and Giora (1997), the SPM suggests that the access to non-literal meaning takes place only after the activation of the corresponding literal meaning and the detection of contextual incongruity. This model predicts that if primed by LC conditions, there will be smaller N400 amplitudes elicited by the Targets, as well as faster responses and higher accuracy rates in the semantic judgment task compared to MC conditions.

Obviously, both the behavioral and the ERP results of the HF items in this study are not in line with this prediction, as they both suggest that the activation of metaphorical meaning of HF Chinese *xiehouyus* was neither slower nor more difficult than that of the literal meaning (*p *> .1 between HFMC and HFLC in ERP and behavioral results). It demonstrates that further inferential processes other than initial access are not necessarily required for a contextually fit interpretation of HF Chinese *xiehouyus* appearing in MC conditions.

As for the LF items, however, the worse performance of judgment in MC conditions than in LC conditions (94.1% vs. 96.8%, *p *= .031) might implicate a harder process of figuring out a metaphorical interpretation, which is in line with the predictions of the SPM. More specifically, the participants might work out the contextually coherent metaphorical interpretation via a two-step mechanism, and to some extent they got stuck at the conceptual mapping processes during the phase of revision and adjustment to contextual information (i.e. the second step) after the literal meaning was accessed (i.e. the first step).

### The Direct Access Model (DAM)

According to Wang (2009), words or sentences can hardly be regarded as metaphorical unless being used in a metaphorically-biasing context, and this is also the case in Chinese *xiehouyus* (Shu 2015). In contrast with the SPM, the DAM proposed by Gibbs (1984) lays much emphasis on the role played by contextual information, and argues that if supported by highly constrained contextual information which is strongly biased in favor of a certain interpretation, the contextually coherent interpretation is able to be tapped directly and exclusively very early on (Giora 2003). In the present study, this model predicts that the activation of metaphorical meaning will probably be observed on the Targets in MC conditions at an early stage.

The N400 effect in the HFMC was smaller than that in HFLC condition (.809 µV vs. − .326 µV, *p *< .001), which seems to support this prediction. However, in the case of LF antecedents, no solid evidence can be found from the experiment to prove the early activation of the metaphorical meaning in MC. Therefore, this model may to some extent account for the activation of the metaphorical meaning of HF Chinese *xiehouyus*, but fails to make sense in the case of LF ones.

### The Graded Salience Hypothesis (GSH)

According to the GSH, two mechanisms run in parallel in language processing: One is bottom-up and sensitive only to domain-specific linguistic information, and another is top-down, sensitive to contextual information. The lexical access mechanism facilitates the automatic activation of salient meaning regardless of contextual information (Giora 2003). Particularly, the salient meaning here refers to the coded meaning stored in people’s mental lexicon, and the saliency of a word is determined by the joint influence of various factors, including familiarity. In the case of non-literal language, if metaphorical meaning is more salient than literal meaning, the former will get activated rapidly during the lexical retrieval stage, while expressions without a salient metaphorical meaning will be processed as literal meaning. Additionally, the separate expectation-driven contextual mechanism along with the lexical access mechanism will operate globally in language comprehension at the point where contextual information has already been processed and interfaced with other cognitive processes (e.g., inferencing) (Peleg et al. 2001).

In this study, this hypothesis seems to well account for the processing of both HF and LF items. As for HF *xiehouyus*, the smaller N400 elicited in the HFMC condition than in the HFLC condition on Target (.809 µV vs. − .326 µV, *p *< .001) indicates a rapid activation of the salient metaphorical meaning of the HF antecedents. Yet, the fact that the discrepancy between N400 elicited in MC and LC conditions disappeared in the Probes (no main effect of context) can be regarded as a reflection of the expectation-driven contextual mechanism, facilitating the activation of the less salient literal meaning, while the salient metaphorical meaning was suppressed. As for the LF items, the participants comprehended them better in LC conditions than in MC conditions (Accuracy: 96.8% vs. 94.1%, *p *= .031). From this we can conclude that the comprehension of LF Chinese *xiehouyus* should be based on a bottom-up meaning construction mechanism rather than on the activation of previously stored non-literal meaning, and that their lexicalized literal meaning would be processed first (Giora 2003: 19).

To sum up, the discussion in this section has examined the prevailing models of non-literal language dynamic processing mechanism. Evidence from the processing of LF Chinese *xiehouyus* seems to support the SPM and GSH, while the processing mechanism of HF items seems to support the DAM as well as the GSH, as both of them successfully predict the rapid access to the metaphorical meaning. Therefore, the GSH can provide a better acceptable explanation for the processing mechanism of Chinese *xiehouyus* varied in familiarity. Additionally, the findings in this reexamination support that non-literal language comprehension is a complicated process influenced by both external and internal factors like context and familiarity.

### Whether the Antecedents of a Chinese *Xiehouyu* Can Be Used Independently

Results from this study also provide insights into the question: Are the antecedents of a Chinese *xiehouyu* able to be used independently? Wen (2005) made an attempt to answer this question by investigating 4893 pieces of Chinese *xiehouyus* in over 520 literature works, and he found only 375 antecedents were used independently, less than 1/12 of the total, demonstrating that only a very small proportion of antecedents appear independently in daily use.

In this study, the behavioral and the ERP results both demonstrate that only HF antecedents can be used independently. As discussed before, HF Chinese *xiehouyus* required much shorter RT (726.404 ms vs. 1161.715 ms, *p* < .001) and got higher accuracy (98.3% vs. 95.5%, *p* < .001) than LF ones in semantic judgment tasks, and the consequents of HF Chinese *xiehouyus* elicited much smaller N400 than LF ones both on Target (− .568 µV vs. − .804 µV, *p* < .001) and Probe (.703 µV vs. .171 µV, *p *< .001), indicating that the anomalous two-part allegorical saying was hardly detected when the participants intended to integrate the consequent to its antecedent. In other words, HF Chinese *xiehouyus* have been stored as chunks so that the consequents can be activated upon encounter of the antecedents through a lexical retrieval process in long term memory. A questionnaire study performed by Shu (2015) also supports this opinion: when HF antecedents were provided, almost all the participants were able to work out the correlating consequents, but they all failed to do it when facing LF antecedents.

Moreover, as stated in the introduction, the metaphorical meaning of Chinese *xiehouyus* is commonly pointed out by the consequents. However, the antecedents of HF Chinese *xiehouyus* can be taken as the vehicles of highly conventional metaphors that bear the intended metaphorical meaning of the whole structure. In this sense, the presence of consequents seems unnecessary since the antecedents have already taken their roles.

As for LF Chinese *xiehouyus*, however, both the robust N400 detected on the Targets and the Probes as well as the significantly longer RT and lower accuracy indicate that the integration of the antecedents and consequents was rather difficult for the participants, as both the form and the semantic relation between the antecedents and the consequents were novel to the participants, and much extra cognitive efforts were required to derive the metaphorical meaning from literal interpretation via conceptual mapping mechanism. During such processes, failures may also occur. Owing to these facts, successful predictions on the consequents from the LF antecedents standing alone can be hardly achieved, and the metaphorical meaning of LF Chinese *xiehouyus* cannot be directly manifested by their antecedents. Consequently, LF antecedents cannot be use independently.

In general, this study agrees with the opinion that only antecedents of HF Chinese *xiehouyus* can be used independently, or at least have high potentials to be used independently, but the LF ones are excluded for the independent use of antecedents. Additionally, in daily communication and literature works, people may prefer to provide the complete structure of Chinese *xiehouyus* out of some pragmatic or rhetorical intentions (Shu 2015).

## Conclusion

This research has examined the online processing mechanism of Chinese *xiehouyus* by ERP technique and has tried to answer the three questions put forward in the introduction. For the first question, we conclude that both familiarity and linguistic context play roles respectively and interactively in Chinese *xiehouyus* processing. Familiarity is a predominant factor for meaning activation and the predictability of consequents. Context can also promote or suppress the meaning access by associating with antecedents. And the interactive effect of the two factors can be explained by the career of metaphor hypothesis. HF *xiehouyus* serve as vehicles of conventional metaphors, whose metaphorical meaning can be directly extracted from the mental lexicon, being promoted by MC conditions. Meanwhile, LF *xiehouyus* are the vehicles of novel metaphors, and their metaphorical meaning has to be deducted from the salient literal meaning, which is accessed more quickly in LC conditions.

Concerning the second question, this study has found that (1) The SPM can account for some evidence from the processing of LF Chinese *xiehouyus*; (2) The DAM can explain the mechanism of HF Chinese *xiehouyus* processing but not of the LF ones; (3) The GSH can account for the processing of Chinese *xiehouyus* in varied familiarity.

The finding for the third question is that only HF antecedents can be used independently, or at least have high potentials to be used independently, but it is not the case for the LF ones. However, in daily communication and literature works, people tend to provide the complete structure of Chinese *xiehouyus* for pragmatic purposes.

## Electronic supplementary material

Below is the link to the electronic supplementary material.

Supplementary material 1 (XLSX 19 kb)

## References

[CR1] Bowdle BF, Gentner D (2005). The career of metaphor. Psychological Review.

[CR2] Gibbs RW (1984). Literal meaning and psychological theory. Cognitive Science.

[CR3] Gibbs RW (2002). A new look at literal meaning in understanding what is said and implicated. Journal of Pragmatics.

[CR4] Giora R (1997). Understanding figurative and literal language: The graded salience hypothesis. Cognitive Linguistics.

[CR5] Giora R (1999). On the priority of salient meanings: Studies of literal and figurative language. Journal of Pragmatics.

[CR6] Giora R (2003). On our mind: Salience, context, and figurative language. Language in Society.

[CR7] Giora R, Fein O (1999). Irony comprehension: The graded salience hypothesis. Humor – International Journal of Humor Research.

[CR8] Gong Y, Zhou R (2009). The career of metaphor and its explanatory power. Foreign Language and Literature (Journal of Sichuan International Studies University).

[CR9] Grice HP, Cole P, Morgan J (1975). Logic and conversation. Syntax and semantics 3: Speech acts.

[CR10] Katz AN, Ferretti TR (2001). Moment-by-moment reading of proverbs in literal and non-literal contexts. Metaphor & Symbol.

[CR11] Kutas M, Federmeier KD (2011). Thirty years and counting: finding meaning in the N400 component of the event-related brain potential (ERP). Annual Review of Psychology.

[CR12] Kutas M, Hillyard S (1980). Reading senseless sentences: Brain potentials reflect semantic incongruity. Science.

[CR13] Lai H (2008). Understanding and classifying two-part allegorical sayings: Metonymy, metaphor, and cultural constraints. Journal of Pragmatics.

[CR14] Lau EF, Namyst A, Fogel A, Delgado T (2016). A direct comparison of N400 effects of predictability and incongruity in adjective-noun combination. Collabra.

[CR15] Lim EAC, Ang SH, Lee YH, Leong SM (2009). Processing idioms in advertising discourse: Effects of familiarity, literality, and compositionality on consumer ad response. Journal of Pragmatics.

[CR16] Luck SJ (2005). An introduction to the event-related potential technique.

[CR17] Ma L, Ma Y, He X, Liu H, Zhang J (2019). Processing of Chinese homophonic two-part allegoric sayings: Effects of familiarity and homophone. Acta Psychologica Sinica (XINLI XUEBAO).

[CR18] Ma L, Zhang J (2011). The internal structure of Chinese *xiehouyu*. Applied Linguistics (YUYAN WENZI YINGYONG).

[CR19] Peleg O, Giora R, Fein O (2001). Salience and context effects: Two are better than one. Metaphor and Symbol.

[CR20] Qu, W. (2010). *The mechanism of Chinese XIEHOUYU comprehension*. Doctoral dissertation, Hunan Normal University.

[CR21] Schweigert WA, Danny R (1988). Familiar idiom comprehension. Journal of Psycholinguistic Research.

[CR22] Searle JR, Searle JR (1979). Literal meaning. Expression and meaning: Studies in the theory of speech and irony processing. Discourse processes, 35, 241–279. acts.

[CR23] Shu D (2015). Chinese *xiehouyu* and the interpretation of metaphor and metonymy. Journal of Pragmatics.

[CR24] Titone D, Libben M (2014). Time-dependent effects of decomposability, familiarity and literal plausibility on idiom priming: A cross-modal priming investigation. The Mental Lexicon.

[CR25] Titone DA, Connine CM (1999). On the compositional and noncompositional nature of idiomatic expressions. Journal of Pragmatics.

[CR26] Wang X (2009). Chinese metaphorical cognition and its ERP imaging.

[CR27] Wang X, Xu C (2008). Agentive and contextual factors affecting metaphorical cognition. Foreign Languages and Their Teaching (WAIYU JIAOXUE).

[CR28] Wang X, Zheng W, Zhao L, Liu Y, Huang B, Zhang X (2018). The role of context in processing Chinese three-character verb–object metaphors: An event-related potential study. Psychological Reports.

[CR29] Wen D (2005). Chinese lexicology.

[CR30] Wen D (2011). Dictionary of Chinese Xiehouyus.

[CR31] Zhang H (2016). Idiom representation and processing: A neurocognitive approach.

[CR32] Zhang H, Jiang L, Gu J, Yang Y (2013). Electrophysiological insights into the processing of figurative two-part allegorical sayings. Journal of Neurolinguistics.

[CR33] Zhang J, Ma L, Zhang J (2018). Processing of Chinese two-part allegorical saying: Effects of familiarity and ISI between front and back parts. Psychological research (XINLI YANJIU).

